# The role of perineural invasion in penile cancer: a meta-analysis and systematic review

**DOI:** 10.1042/BSR20180333

**Published:** 2018-10-31

**Authors:** Xiang Zhou, Feng Qi, Ruhua Zhou, Shangqian Wang, Yamin Wang, Yi Wang, Chen Chen, Yichun Wang, Jie Yang, Ninghong Song

**Affiliations:** 1 Department of Urology, First Affiliated Hospital of Nanjing Medical University, No. 300, Guangzhou Street, Nanjing, Jiangsu Province 210029, China; 2 College of Nursing, Nanjing Medical University, No. 101, Longmian Street, Nanjing, Jiangsu Province 210029, China

**Keywords:** meta-analysis, penile cancer, perineural invasion, systematic review

## Abstract

The significance of perineural invasion (PNI) present in penile cancer (PC) is controversial. In order to clarify the predictive role of PNI in the inguinal lymph node (ILN) metastases (ILNM) and oncologic outcome of patients, we performed this meta-analysis and systematic review. The search of PubMed, Embase, and Web of Science was conducted for appropriate studies, up to 20 January 2018. The pooled odds ratio (OR) and hazard ratio (HR) with their 95% confidence interval (CI) were applied to evaluate the difference in ILNM and oncologic outcome between patients present with PNI and those who were absent. A total of 298 in 1001 patients present with PNI were identified in current meta-analysis and systematic review. Significant difference was observed in ILNM between PNI present and absent from patients with PC (OR = 2.98, 95% CI = 2.00–4.45). Patients present with PNI had a worse cancer-specific survival (CSS) (HR = 3.58, 95% CI = 1.70–7.55) and a higher cancer-specific mortality (CSM) (HR = 2.20, 95% CI = 1.06–3.82) than those cases without PNI. This meta-analysis and systematic review demonstrated the predictive role of PNI in ILNM, CSS, and CSM for PC patients.

## Introduction

Penile cancer (PC), as a rare malignancy, was estimated to affect approximately 2000 Americans in 2017 [1]. However, In South America, Southeast Asia, and parts of Africa, the incidence of PC reaches up to 10–20% in male malignancies [2]. Pathologically, squamous cell carcinoma (SCC) is the predominant subtype of PC, and rare clear cell carcinoma along with extramammary Paget disease (EMPD) of penis had also been described [3–6]. Clinically, the lymph node metastasis (LNM) always indicates a poor prognosis. And, 5-year survival rate may diminish to 80% in patients with inguinal lymph nodes (ILNs) metastases (ILNM), 0–33% with pelvic lymph nodes (PLNs) involvement [7,8].

Traditionally, radical ILN dissection (ILND) is standard-of-care for patients with positive ILNs, which can significantly improve the outcome of 80% patients with low-volume positive ILNs [9]. Unfortunately, radical ILND leads to approximately 40–70% of patients suffering major or minor complications. Some studies indicated that 30–50% of patients with clinically positive ILNs were treated based on ‘risk factors’ provided by guidelines, such as clinical ILNs status, tumor grade and stage [10,11]. Meanwhile, occult metastases exist in approximately 25% patients with non-palpable nodes and surgical lymph node staging proposed for intermediate (pT1G2) and high risk patients (any T2 or grade 3–4) according to the 2014 European Association of Urology (EAU) guidelines undoubtedly increases the medical expenses and suffering of PC patients [12,13].

Although some pathologic factors predicting LNM and prognosis of PC, including tumor grade, stage, lymphovascular invasion, LNM, and extranodal extension, were widely accepted and included in the 8th AJCC TNM staging, the outcomes predicted by them are not very accurate [10–12]. Many other risk factors, such as subtypes of PC, the thickness and depth of neoplasm, site, patterns of growth and invasion, perineural invasion (PNI), and positive margins of resection, were also found to hold predictive value in PC, but results from studies were discordant. So, these pathologic factors were limited in clinical practice.

PNI in PC, defined by Velazquez et al. [14] that the perineural space was open and clear, and the tumor invaded outside the perineural room, was first introduced in 2009 EAU Guidelines within many other potential pathologic risks for LNM [15]. However, it did not acquire sufficient attention even in the 2014 EAU Guidelines and 2017 National Comprehensive Cancer Network (NCCN) Guidelines until in the updates in 8th AJCC TNM staging guideline. The PNI was included to divide T1a and T1b, suggesting PNI was a significantly risk factor of LNM and outcome of patients [16]. Nevertheless, PNI still is a controversial predictor in PC patients. Several studies indicated that PC patients with PNI carried a higher risk of ILNM [14,17–19]. On the contrary, several researchers took an opposite opinion [5,20,21]. Meanwhile, limited studies demonstrated PNI as an independent prognostic factor in multivariate analysis. Hence, in order to evaluate its predictive role in prognosis and assess the relationship between PNI and ILNM, a meta-analysis and systematic review was performed by including all eligible studies to distinguish this statistical evidence.

## Materials and methods

### Search strategy

**Figure 1:**
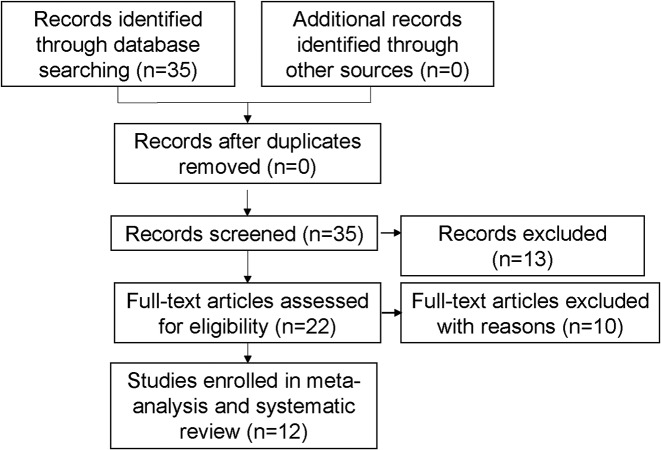
Study selection process

A systematic research was conducted on PubMed, Embase, and Web of Science for potential articles, according to ‘PRISMA’ guideline (till 20 January 2018) [22]. The following medical subjects and combinations were applied: (‘PC’, ‘penile neoplasms’, ‘penile cancer’, ‘penile carcinoma’ or ‘the squamous of penis’), (‘perineural invasion’, ‘perineural infiltration’, or ‘PNI’). Furthermore, we also manually screened the reference list of additional papers. A flow diagram of the research selection process is illustrated in Figure 1.

### Inclusion criteria and exclusion criteria

Studies included had to meet the following criteria: (i) retrospective studies detailing lymph nodes state and outcome of PC patients, (ii) patients were pathologically diagnosed with PC with or without PNI, and (iii) sufficient data could be extracted from included articles. Conversely, the exclusion criteria were as follows: (i) no available or complete data found in included studies, (ii) non-original researches, and (iii) overlapped data appearing in studies.

### Data extraction

All worthy data extracted from eligible studies were identified by two independent researchers (X.Z. and F.Q.). The examination of results was undertaken by two investigators (R.Z. and S.W.). The useful data from each study were recorded as follows: age, follow-up period, number of patients, pathologic subtypes, number of PNI patients, number of ILND patients, number of ILNM patients, and hazard ratio (HR)/odds ratio (OR) with 95% confidence intervals (CI) of oncologic outcome. Moreover, the quality of all eligible studies were evaluated by Newcastle–Ottawa Scale.

### Statistical analysis

Stata software (version 12.0; Stata Corp LP, College Station, TX) was applied to statistical analysis. The association of PNI with ILNM in patients with PC was assessed by ORs with 95% CIs. For analyzing the role of PNI in outcome of PC patients, the HR and its 95% CI calculated by raw data supplied in article or directly reported by included researches were synthesized, and when HR or its 95% CI was not directly offered, the method described by Altman and Bland [23] was adapted to calculate one of them based on its *P*-value. If authors offered the results of multivariable and univariable analyses, we recognized the former. Heterogeneity assumption was verified by Galbraith plot, and when heterogeneity appeared, it was more appropriate to adopt the random-effects model other than fixed-effects model to estimate the pooled ORs/HRs. Additionally, a sensitivity analysis was conducted to confirm the robustness of the pooled results, during which data from each individual study was sequentially removed. Moreover, Begg’s funnel plot was conducted to evaluate the publication bias between the studies [24]. Two-tailed *P*-values were considered statistically significant when less than 0.05.

## Results

### Studies’ characteristics

A total of 1001 PC patients, 298 patients present with PNI, were identified in current meta-analysis and systematic review for a future evaluation [5,6,14,17–21,25–28]. In addition to SCC patients, there were 41 cases with EMPD eligible for analysis. Those whose age ranged from 52 to 88 years were treated between 1980 and 2016. In these 12 included studies, PC patients came from America [19], U.K. [28], Australia [5], China [18,26], Brazil [14,17,19,21], and Paraguay [14,19]. In addition, the detailed characteristics of enrolled studies were summarized in Table 1 [5,6,14,17–21,25–28]. The association between PNI and ILNM was reported by eight studies [5,14,17–21], but one study was excluded from this meta-analysis because of limited data [6]. Another two studies evaluated the role of PNI in cancer-specific survival (CSS) and cancer-specific mortality (CSM) of patients, listing HR and its 95% CI in multivariable Cox regression, respectively. Moreover, the HR or 95% CI of CSS and CSM was calculated in two articles according to the method described by Altman and Bland [23] based on *P*-value and HR/95% CI [23,26,27]. Additionally, there were two authors providing the raw data of articles, so we calculated the CSS and overall survival (OS) with HR along with their 95% CI in one study, and OS with HR and 95% CI in another article, using multivariable Cox regression [20,17]. Shu et al. [18] and Sanchez et al. [6] described the number of deaths in patients with and without PNI, which were not applied to meta-analyses for outcome of patients.

**Table 1:** Detailed characteristics of the studies included in this meta-analysis and systematic review

First author	Year	Country	Recruitment period	Study design	Age (mean/median)	Follow-up (mean/ median)	No. pts	Pathology	No. PNI pts	No. ILNM pts		Outcomes		NOS
										PNI (P) group	PNI (A) group	PNI (P) group death	PNI (A) group death	
**Ornellas** [21]	2008	Brazil	1996–2007	Retrospective	57	74	196	SCC	44	23	47	NR		8
**Velazquez** [14]	2008	Brazil + Paraguay	NR	Retrospective	Mean: 55	NR	134	SCC	48	33	33	NR		8
**Chaux** [19]	2010	U.S.A. + Paraguay + Brazil	NR	Retrospective	Mean: 62	85	45	SCC	10	6	6	NR		7
**Gonzaga-Silva** [17]	2012	Brazil	1999–2010	Retrospective	Mean: 65	31.9	16	SCC	11	3	1	OS:HR 0.462, 95% CI (0.180–33.618)		6
**Mannweiler** [5]	2013	Austria	1993–2013	Retrospective	NR	47	76	SCC	11	6	6	2	NR	7
**Mentrikoski** [20]	2014	U.S.A.	1990–2012	Retrospective	62	20	59	SCC	25	12	14	CSS:HR 5.287, 95% CI (0.484–57.752); OS:HR 2.424, 95% CI (0.790–7.439)		8
**Vassallo** [25]	2015	Brazil	NR	Retrospective	52	NR	122	SCC	48	NR		CSS:HR 2.47, 95% CI (0.9840–6.2078); DFS:HR 2.78, 95% CI (0.9628–8.0687)		6
**Shu** [18]	2015	China	2004–2013	Retrospective	65	36	41	EMPD	5	4	10	2	11	6
**Geng** [26]	2015	China	1997–2009	Retrospective	66.5	52	43	SCC	8	NR		CSS:HR 8.24, 95%CI (1.8306–37.09)		6
**Sanchez** [6]	2016	Paraguay	NR	Retrospective	88	12	3	SCC	3	1	NR	2	0	8
**Cunha** [27]	2016	Brazil	1980–2014	Retrospective	55	73	149	SCC	40	NR		CSM:HR 1.879, 95% CI (0.945–3.736); ACM:HR 1.553, 95% CI (0.901–2.676)		7
**Albersen** [28]	2017	U.K.	2005–2016	Retrospective	61.9	33.7	117	SCC	45	NR		LR: HR 2.9713, 95% CI (0.8154–10.8276); CSM:HR 3.1615, 95% CI (0.5604–17.8371)		7

Abbreviations: ACM, all cause mortality; DFS, disease-free survival; LR, local recurrence; NOS, Newcastle–Ottawa Scale; NR, not reported; PNI(A), PNI (absent); PNI(P), PNI (present); pts, patients.

### ILN metastases

**Figure 2:**
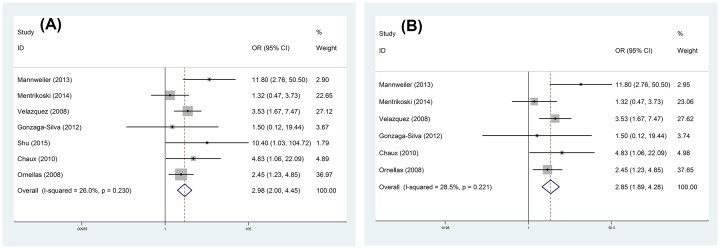
Forest plots of predictive role of PNI in ILNM for PC: (A) various kinds of PC and (B) SCC of PC.

**Figure 3:**
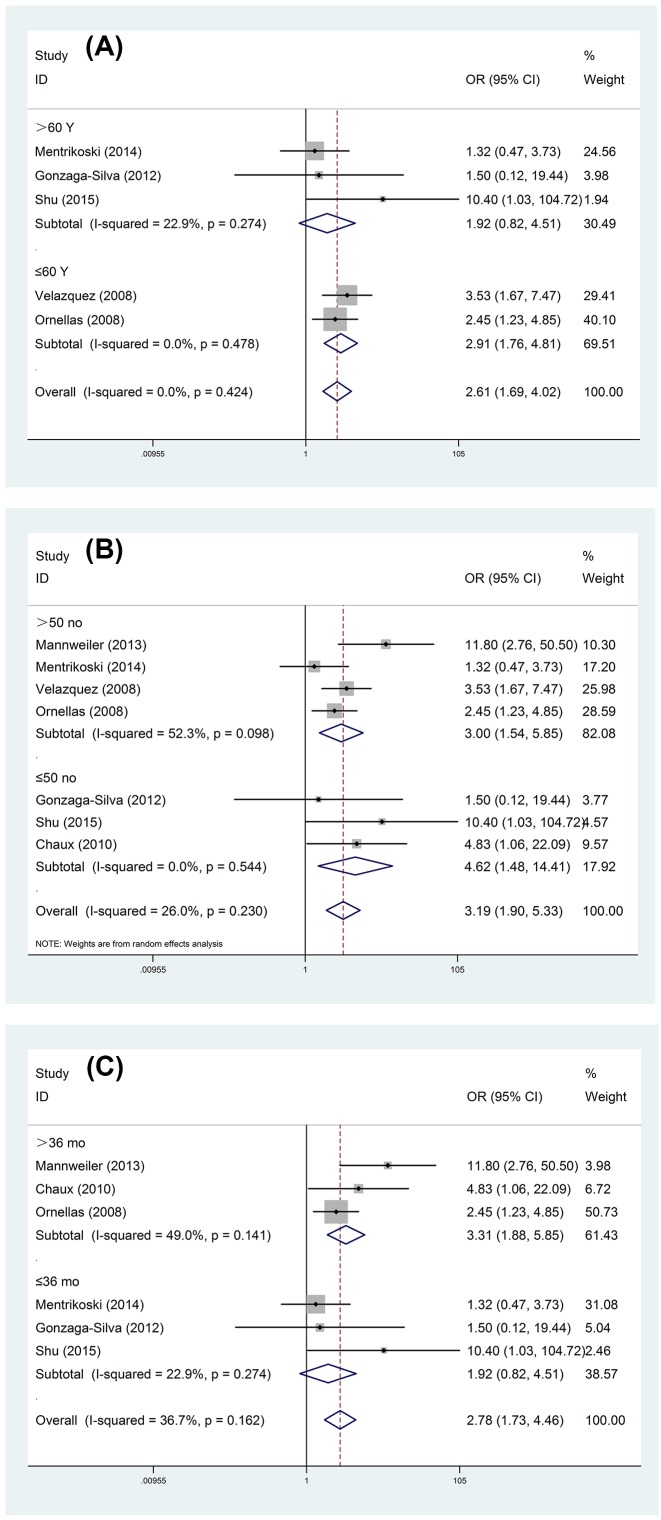
Forest plots of stratified analysis of the role of PNI in ILNM: (A) age of patients; (B) sample size of study; (C) follow-up time of reaearch.

Significant difference was observed in ILNM between PNI present and absent from patients with PC (OR = 2.98, 95% CI = 2.00–4.45) (Figure 2A) after seven studies were synthesized, and patients with PNI undertook a higher risk of ILNM. For PNI in SCC, the main subtype of PC, the risk of ILNM was also apparent (OR = 2.85, 95% CI = 1.89–4.28) (Figure 2B). Meanwhile, we carried out subgroup analysis according to the age of patients, follow-up time, and sample size (Table 2 and Figure 3). Our meta-analysis demonstrated that the predictive value of PNI in ILNM only showed in studies with patients younger than 60 years old (OR = 2.91, 95% CI = 1.76–4.81) and follow-up time longer than 36 months (OR = 3.31, 95% CI = 1.88–5.85).

**Table 2:** Detailed result of stratified analysis of the role of PNI in ILNM according to the age of patients, sample size of study, and follow-up time of research

Analysis	Number of studies (number of patients)	OR (95% CI)	*P*	Model	Heterogeneity	
					*I^2^* (%)	*P_het_*
**Subgroup 1**						
Age (years)						
≤60	2 (330)	2.91 (1.76–4.81)	0.000	Fixed	0	0.478
>60	3 (116)	1.92 (0.82–4.51)	0.134	Fixed	22.9	0.274
**Subgroup 2**						
Sample size (no)						
≤50	3 (102)	4.62 (1.48–14.41)	0.008	Random	0	0.544
>50	4 (465)	3.00 (1.54–5.85)	0.001	Random	52.3	0.098
**Subgroup 3**						
Follow-up time (months)						
≤36	3 (116)	1.92 (0.82–4.51)	0.134	Fixed	22.9	0.274
>36	3 (317)	3.31 (1.88–5.85)	0.000	Fixed	49	0.141

Abbreviation: no, number.

### Oncologic outcomes of PC patients

**Figure 4:**
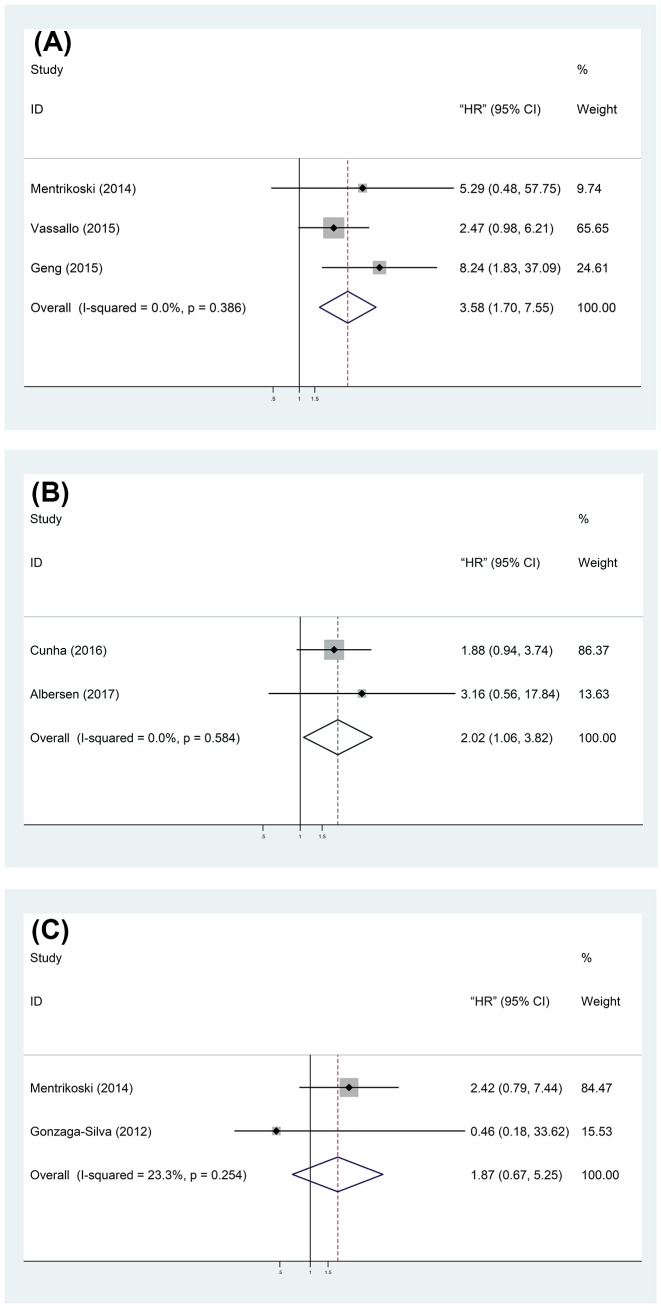
Forest plots of the association between PNI and prognosis: (A) CSS; (B) CSM; (C) OS.

Disease-free survival (DFS), all cause mortality (ACM), or local recurrence (LR) was respectively reported in single study to assess the relationship between PNI and outcome of PC patients, which were not used for outcome analysis. Our result showed that patients present with PNI held a worse CSS (HR = 3.58, 95% CI = 1.70–7.55) (Figure 4A) and a higher CSM (HR = 2.02, 95% CI = 1.06–3.82) (Figure 4B) than those without PNI. However, no significant difference was observed in OS of patients with or without PNI (HR = 1.87, 95% CI = 0.67–5.25) (Figure 4C).

### Test of heterogeneity、sensitivity and publication analysis

No obvious heterogeneity was demonstrated. The pooled OR for ILNM (*P*=0.230 for PC, *P*=0.221 for SCC), and pooled HR for CSS (*P*=0.386), CSM (*P*=0.584), OS (*P*=0.254) were conducted by utilizing fixed-effort model.

The sensitivity analysis again confirmed the low heterogeneity of the whole studies.

**Figure 5:**
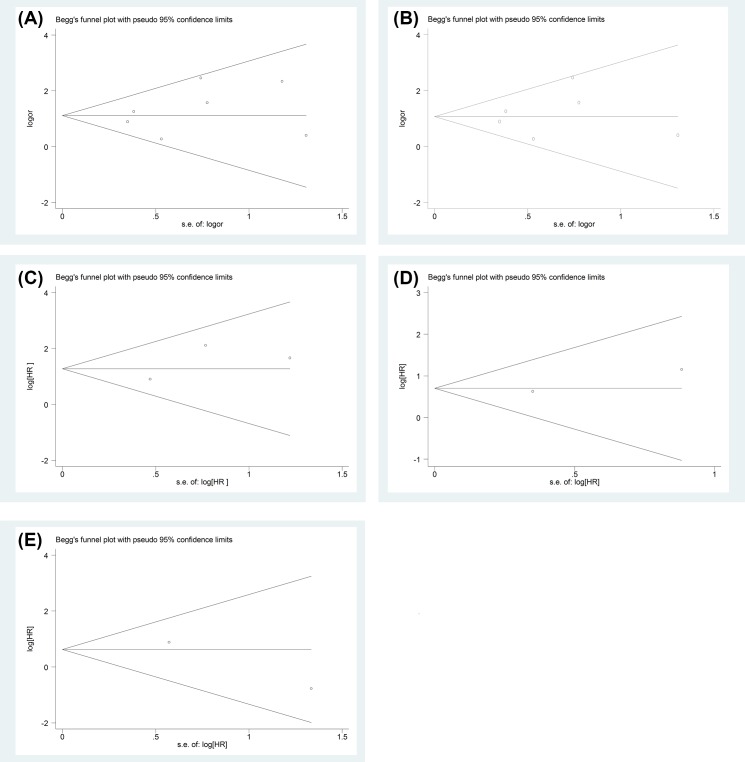
Funnel plot for all studies included in this meta-analysis Funnel plot assessing predictive role of PNI in ILNM for various kinds of PC ((**A**) Begg’s test, *P*=0.548) and SCC ((**B**) Begg’s test, *P*=0.707) and association between PNI and CSS ((**C**) Begg’s test, *P*=1), CSM ((**D**) Begg’s test, *P*=1) or OS ((**E**) Begg’s test, *P*=1).

Begg’s funnel plot was also used to evaluate the publication bias of the studies, and the shape had no evidence of obviously asymmetrical, no significant publication bias appearing (Figure 5). Hence, the outcome of this meta-analysis was statistically robust.

## Discussion

Amongst all of the PC patients, approximately 15% of the cases are less than 50 years old [29]. Traditional radical ILND leads to considerable complications. In fact, nearly 50% of patients with palpable nodes and those 75% with clinical negative lymph nodes could be free from the lymph node dissection and concomitant morbidities [10]. For prognosis, LNM and the distant metastases are the most significant predictors [13]. PC patients with pN1 could be able to have 79–89% of 5-year CSS, 7–60% for pN2 and 0–7% for pN3 [30]. Obviously, the difference in CSS is visible even in same risk group. Accordingly, investigating novel pathological predictors of LNM and prognosis is urgent and necessary in adoption of appropriate treatments, and PNI newly incorporated in the newest 8th AJCC TNM staging is promising.

There are small branches of peripheral nerves discovered in Buck’s fascia, preputial dartos, and corpus spongiosum. It is apparent of morphologic discrimination of PNI and not difficult to identify this lesion [31]. Nonetheless, the similarity between PNI and nerve trapped in tumor deserves more attention. The predictive role of PNI for ILNM in SCC invading 5–10 mm was first well demonstrated in a multicenter retrospective study by Velazquez et al. [14] with multivariate logistics regression models in 2008. On contrast, Ornellas et al. [21] from Brazil failed to find that PNI was an independent predictor for ILNM in the same year. The reason for these two contradicted consequence may lie in small number of cases, various therapeutic methods, and heterogeneous pathologic methodologies, which highlight the necessity of meta-analysis in PNI.

The result of this meta-analysis demonstrated that patients with PNI take a considerable risk of carrying ILNM. A subgroup analysis classified by sample size was performed, regardless of the number of each studies, we found statistical significance between PNI and ILNM. These data could be partly explained by that PNI exists in a considerable portion of PC individuals, 30% in our analysis, and can be identified in tumor mass without difficulty. Moreover, association between PNI and ILNM with significance was merely observed in studies with mean/median follow-up time no less than 36 months, which may be attributed to increased ILNM rate appearing during longer follow-up. Additionally, an interesting and crucial finding with regard to the impact of age in the predictor of PNI for ILNM revealed by stratified analysis, our analysis suggested that when PC patients who were younger than 60 years were present with PNI, the hazard of developing ILMN was apparent and statistically significant compared with individuals older than 60 years. This consequence was consistent with result depicted by Paiva et al. [32] that younger patients took more aggressive behavior of PC, which should receive much attention.

Because of the small number of previous studies, there is no consensus on the PNI and relationship with CSS. This meta-analysis could be able to overcome the shortcomings of single study, limited sample size, and insufficient statistical power, it showed that PC patients with PNI undertook adverse CSS and CSM, but not OS.

The early detection of metastases in lymph nodes is promising. Dynamic sentinel node biopsies (DSNB) has been recommended for clinical node negative patients with intermediate and high-risk stage in Europe [33], and sensitivity reached up to 88 and 90% with use of patent blue in a meta-analysis [13]. Nevertheless, the high expenses precludes its implementation in developing countries. Besides, fine needle aspiration cytology (FNAC) with ultrasound (US) can detect tumor in palpable nodes excellently, rapidly, and easily [9,13]. However, when results of pathology are negative, the option of following procedure is in an embarrassing state. Moreover, the sensitivity of conventional radiological examination, such as US, computed tomography (CT) and MRI, for detecting metastases in ILNs is disappointing [34–36]. Recently, positron emission tomography (PET)/CT, a promising radiological examination, demonstrated excellent specificity and sensitivity for distinguishing metastases in palpable nodes of 100 and 90% repactively, whose utility is restricted by limited reliable evidence [37]. Additionally, lymphotrophic nanoparticle-enhanced MRI (LNMRI) showed the promise of implementation, which needs further follow-up [38]. Hence, PNI plays an important role in predicting metastases in lymph nodes and outcome of PC patients in this situation.

Admittedly, this meta-analysis also had some limitations. First, only 12 relevant researches were included, therefore, higher quality and large sample size clinical studies are needed to strengthen our conclusion, especially in the association between prognosis and PNI. Second, some data of eligible studies published between 1980 and 2016, existing different diagnostic criteria and standards of treatment, which increased bias of this meta-analysis. Third, we estimated HR or 95% CI with method described by Altman and Bland [23], which were not same as reported by authors.

## Conclusion

The results of this meta-analysis demonstrated that PNI is a worthy predictor of ILNM and imply a poorer CSS and higher CSM in PC patients. Moreover, attention ought to be given to those patients present with PNI who are younger than 60 years. And, higher quality and large sample size clinical studies are needed to strengthen our conclusion

## Abbreviations

CI: confidence interval
CSM: cancer-specific mortality
CSS: cancer-specific survival
CT: computed tomography
EAU: European Association of Urology
EMPD: extramammary Paget disease
HR: hazard ratio
ILN: inguinal lymph node
ILND: ILN dissection
ILNM: ILN metastasis
OR: odds ratio
OS: overall survival
PC: penile cancer
PNI: perineural invasion
US: ultrasound
